# Hf(OTf)_4_-Catalyzed Three-Component Synthesis of *N*-Carbamate-Protected β-Amino Ketones

**DOI:** 10.3390/molecules27031122

**Published:** 2022-02-08

**Authors:** Zhen-Zhen Chen, Dong-Zhao Yang, Ying-Ying Dong, Mei Chi, Shou-Zhi Pu, Qi Sun

**Affiliations:** 1 Jiangxi Key Laboratory of Organic Chemistry, Jiangxi Science and Technology Normal University, Nanchang 330013, China; chenzhenzhen5@126.com (Z.-Z.C.); yangdongzhao1@126.com (D.-Z.Y.); dongyingying0@126.com (Y.-Y.D.); chimei5@126.com (M.C.); 2 Department of Ecology and Environment, Yuzhang Normal University, Nanchang 330103, China

**Keywords:** Hf(OTf)_4_, Mannich base, three-component reaction, carbamate, reaction mechanism

## Abstract

Hafnium(IV) triflate (Hf(OTf)_4_) has been identified as a potent catalyst for the direct three-component synthesis of β-carbamate ketones. This new method, featuring a low catalyst loading, fast reaction rate, and solvent-free conditions, provided facile access to a diversity of carbamate-protected Mannich bases. A mechanistic investigation indicated that the three-component reaction proceeds via sequential aldol condensation and aza-Michael addition, but not the Mannich-type pathway.

## 1. Introduction

β-Amino ketones, commonly known as Mannich bases, represent an important class of synthetic scaffold [1,2,3,4,5] that has been widely employed in the synthesis of pharmaceutical agents [6,7,8] and natural products [9,10]. Without a doubt, the Mannich reaction [11] is one of the most effective approaches to synthesize β-amino carbonyl compounds. It is widely accepted that the mechanism of the Mannich reaction involves the condensation of aldehyde and amine and the subsequent addition of ketone [1,2,3,4,5]. Currently, a huge number of synthetic methods have been reported for the catalyzed three-component Mannich reaction based on arylamine and alkylamine substrates [12,13,14]. Among various Mannich bases, β-carbamate ketones are of high synthetic value, because the facile removal of carbamate protecting groups offers easy access to β-primary amino ketones. However, it has been reported that the replacement of amine substrates with less reactive carbamates under the same Mannich reaction conditions typically results in significantly lowered yields [15,16].

To date, only a few catalytic systems, including AuCl_3_-PPh_3_ [17], Bi(OTf)_3_ [15], I_2_ [18], FeCl_3_·6H_2_O/Me_3_SiCl [19], H_3_PW_12_O_40_ [20], Me_2_S^+^BrBr^−^ [21], and PPh_3_Br_2_ [22], have been developed to enable direct one-pot, three-component synthesis of β-carbamate ketones from aldehydes, ketones, and carbamates. However, these methods are limited by drawbacks, such as slow reaction rate, low to moderate yields, high catalyst loading, and use of stinky, corrosive, or expensive catalysts. Whilst the catalytic mechanisms of Me_2_S^+^BrBr^−^ and PPh_3_Br_2_ have not been studied, those of the other methods have simply been described as Mannich-type reactions without substantial evidence regarding the formation of key aldimine intermediates. Since it is known that aldimine is extremely hard to form directly from aldehyde and carbamate [23,24], the proposed Mannich reaction-based mechanisms of these reactions are disputable. The true underlying reaction pathways of these carbamate-based three-component reactions still need to be elucidated.

In our previous research, we found that hafnium(IV) triflate (Hf(OTf)_4_) is a highly potent Lewis acid catalyst for many reactions involving the activation of carbonyl [25], such as the selective anomeric deacetylation of peracetylated saccharides [26], the Biginelli reaction [27], and the synthesis of fluorinated benzimidazolines [28] and pyrimido[2,1-*b*][1,3]benzothiazoles [29,30]. Our recent identification of Hf(OTf)_4_ as a highly reactive catalyst for the Mannich reaction [31] inspired our further exploration of its application in the one-pot synthesis of β-carbamate-protected Mannich bases. Herein, we report the development of a novel Hf(OTf)_4_-catalyzed three-component reaction of aldehydes, ketones, and carbamates for the efficient synthesis of β-carbamate ketones. An experiment-based mechanistic investigation was also conducted to reveal the underlying mechanism of this three-component reaction.

## 2. Results and Discussion

In the preliminary experiments, we tested the catalytic reactivity of a series of Lewis acids of Group IVB transition metals. The reaction of 1.0 equiv of acetophenone, 1.0 equiv of benzaldehyde, and 1.5 equiv of benzyl carbamate (Cbz-NH_2_) in CH_3_CN was performed in the presence of 10 mol% of Lewis acid catalyst at ambient temperature. The results listed in Table 1 showed that most of these catalysts, including ZrCl_4_, bis(cyclopentadienyl)zirconium dichloride (ZrCp_2_Cl_2_), ZrOCl_2_, Zr(NO_3_)_4_, ZrO(NO_3_)_2_, hafnium acetylacetonate (Hf(acac)_4_), and bis(cyclopentadienyl)hafnium dichloride (HfCp_2_Cl_2_), were ineffective in this one-pot, three-component synthesis of β-carbamate ketone **1**. Only Hf(OTf)_4_ and HfCl_4_ exhibited moderate catalytic activity. However, this is significantly lower than that observed in the Mannich reaction under similar conditions [31].

In the following research, the effects of solvent on the Hf(OTf)_4_-catalyzed three-component synthesis of **1** were explored. The experimental results (Table 2, Entry 1–4) showed that Hf(OTf)_4_ exhibited similar moderate catalytic activities in CH_2_Cl_2_, toluene, and THF with an even slower reaction rate, whereas Hf(OTf)_4_ was totally ineffective in DMF. Interestingly, under solvent-free conditions, the reaction time was remarkably shortened to 8 h and the yield of **1** was improved to 82%. The elevation of reaction temperature from 20 to 80 °C further shortened the reaction time to 30 min and increased the yield to 91%. However, the yield of **1** dropped when the temperature increased to 100 °C due to the generation of polar by-products (Table 2, Entry 6–7). In addition, the amount of Hf(OTf)_4_ catalyst was also optimized. The experimental results showed that when as little as 2 mol% Hf(OTf)_4_ was used, the reaction could still afford **1** in an excellent yield (90%) within 2 h. However, further reducing the amount of Hf(OTf)_4_ to 1 mol% caused an incomplete reaction and a drop in yield. In a control experiment, when Hf(OTf)_4_ was replaced by TfOH, the reaction under the same conditions only afforded a trace amount of **1**, confirming the catalytic role of Hf(OTf)_4_.

With the optimized conditions, the substrate scope of the Hf(OTf)_4_-catalyzed direct synthesis of β-carbamate ketones was explored. The data in Table 3 showed that this new approach tolerated a variety of substituents on both acetophenone and benzaldehyde substrates at different positions. It also maintained a good catalytic efficacy in the synthesis of ethyl carbamate products. As shown in Table 3, a range of β-carbamate carbonyl compounds **1**–**20** were obtained in 75–91% yields in 2–4 h under solvent-free conditions. It was noted that the reactions with *p*-nitrobenzaldehyde were relatively slower and lower-yielding. This is in accordance with the previous reports that nitro-containing benzaldehydes are typically less reactive substrates in this three-component reaction [17,19,20,32].

**Scheme 1:**
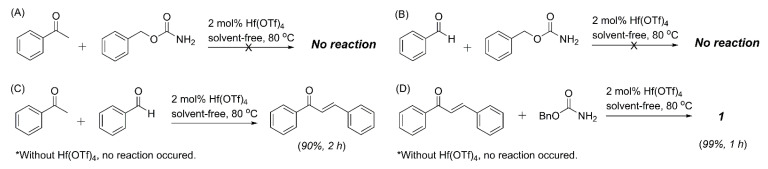
Mechanistic investigation results of the bimolecular reactions of Cbz-NH_2_ with acetophenone (**A**) and benzaldehyde (**B**), acetophenone with benzaldehyde (**C**), and chalcone with Cbz-NH_2_ (**D**). * denotes the control experiment results without Hf(OTf)_4_ catalyst.

**Scheme 2:**
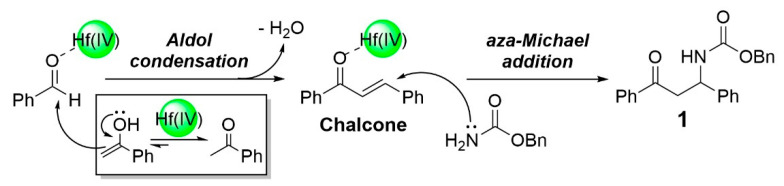
Proposed reaction mechanism for Hf(OTf)_4_-catalyzed three-component reaction.

Multicomponent reactions (MCRs), which employ three or more reactants to yield a product derived from all the starting materials in a one-pot manner, have long been recognized as a powerful tool to enrich molecular diversity in natural product synthesis and medicinal chemistry [33,34,35]. However, the revelation of the reaction mechanisms of MCRs, which is crucial for condition optimization and the manipulation of stereochemistry outcomes, has always been a challenging task due to the multiple potential reaction pathways and the difficulty of capturing key intermediates [35,36,37]. To elucidate the reaction pathway for the three-component synthesis of β-carbamate ketones, we separately tested the reactivity of each component to another under solvent-free conditions. As shown in Scheme 1A,B, Cbz-NH_2_ does not react with either acetophenone or benzaldehyde alone in the presence of 2 mol% of Hf(OTf)_4_ at 80 °C, which excluded the possibility of a Mannich-type reaction mechanism. On the other hand, simply heating benzaldehyde and acetophenone at 80 °C without Hf(OTf)_4_ caused no reaction (Scheme 1C). However, the addition of Hf(OTf)_4_ dramatically promoted the aldol condensation to afford chalcone in a high yield (90%, 2 h). This result is in agreement with the precedents of metal Lewis acid-catalyzed aldol condensation [38,39,40]. In the next stage, we tested the reaction between Cbz-NH_2_ and chalcone in the presence or absence of Hf(OTf)_4_ (Scheme 1D). The experimental results showed that the two components did not react in the absence of a catalyst. In contrast, Hf(OTf)_4_ catalyzed the highly efficient conversion to **1** (99%, 1 h). This result is supported by the previous reports on the catalytic aza-Michael addition of carbamates to enones [32,41,42,43]. Taken together, it is proposed that the Hf(OTf)_4_-catalyzed three-component reaction proceeds via sequential aldol condensation and aza-Michael addition to afford the desired β-carbamate ketones (Scheme 2). Hf(OTf)_4_ exerts catalytic effects on both steps. This is perhaps because the Hf(IV) strongly activates the carbonyl-containing substrates, including benzaldehyde, acetophenone, and chalcone, in both steps (Scheme 2), as has been demonstrated in previous research [27,31,32,44].

## 3. Materials and Methods

### 3.1. General Methods

Chemical reagents and solvents were purchased from Leyan-Shanghai Haohong Scientific Co., Ltd., Shanghai, China. Reactions were monitored by TLC plates coated with 0.25 mm silica gel 60 F_254_ and visualized by UV irradiation (254 nm). Flash column chromatography employed silica gel (particle size 32–63 μm, Qingdao Haiyang Chemicals, Qingdao, China). NMR spectra were acquired on an AV-400 instrument (Bruker BioSpin, Faellanden, Switzerland) with chemical shifts reported in ppm and referenced to CDCl_3_. IR spectra were obtained with a Vertex-70 instrument (Bruker Optics, Billerica, MA, USA). High-resolution mass spectra were obtained with a Dalton micrOTOF-Q II spectrometer (Bruker Optics, Billerica, MA, USA) and reported as *m*/*z*. Melting points were determined with an X-4 digital melting point apparatus and uncorrected (Tech Instrument, Beijing, China). The characterization data of known compounds **1**–**9**, **11**, and **13**–**17** are in agreement with previous reports [17,18,20,23,32,45] and listed in the Supplementary Materials. The characterization spectra of new compounds **10**, **12**, **18**–**20** are also provided in the Supplementary Materials.

### 3.2. General Synthetic Procedure and Characterization of β-Carbamate Ketones

Hf(OTf)_4_ (0.04 mmol, 0.02 eq.) was added to a mixture of aldehyde (2 mmol, 1.0 eq.), ketone (2 mmol, 1.0 eq.), and carbamate (3 mmol, 1.5 eq.) in an open glass vial (10 mL). The reaction was stirred at 80 °C in an aluminum heating module for 2–4 h. The reaction mixture was cooled, dissolved in CH_2_Cl_2_ (1 mL), and loaded on a silica gel column. Flash column chromatography (PE/EA = 5:1) afforded products **1**–**20** in pure form.

Benzyl (3-(4-chlorophenyl)-3-oxo-1-(*o*-tolyl)propyl)carbamate (**10**): a white solid, mp: 108–109 °C. ^1^H NMR (400 MHz, CDCl_3_): *δ* 7.82 (d, *J* = 7.9 Hz, 2H), 7.41–7.26 (m, 8H), 7.18–7.11 (m, 3H), 5.58–5.44 (br, 2H), 5.14–5.03 (m, 2H), 3.61 (d, *J* = 14.8 Hz, 1H), 3.42 (d, *J* = 14.8 Hz, 1H), 2.44 (s, 3H); ^13^C NMR (100 MHz, CDCl_3_): *δ* 196.7, 155.7, 140.0, 139.3, 136.5, 135.8, 135.2, 131.0, 129.7 (×2), 129.1 (×2), 128.7 (×2), 128.3, 128.2, 127.8, 126.6, 125.5, 67.0, 48.5, 43.8, 19.5; IR (KBr): *v*_max_ 3422, 3064, 3031, 2951, 1692, 1588, 1570, 1523, 1455, 1400, 1340, 1252, 1177, 1136, 1092, 1043, 1013, 989, 899, 832, 755 cm^−1^; HRMS (ESI+): *m*/*z* calcd for C_24_H_23_ClNO_3_ [M + H]^+^ 408.1361; found 408.1365.

Benzyl (3-oxo-1-(*o*-tolyl)-3-(*p*-tolyl)propyl)carbamate (**12**): a white solid, mp: 72–74 °C. ^1^H NMR (400 MHz, CDCl_3_): *δ* 7.79 (d, *J* = 7.9 Hz, 2H), 7.38–7.32 (m, 6H), 7.23 (d, *J* = 7.9 Hz, 2H), 7.19–7.12 (m, 3H), 5.68 (br, 1H), 5.58–5.50 (m, 1H), 5.11–5.03 (m, 2H), 3.67–3.49 (m, 1H), 3.49–3.27 (m, 1H), 2.46 (s, 3H), 2.40 (s, 3H); ^13^C NMR (100 MHz, CDCl_3_): *δ* 197.5, 155.7, 144.3, 139.8, 136.6, 135.7, 134.5, 130.9, 129.5 (×2), 128.7, 128.6, 128.4 (×2), 128.3, 128.2 (×2), 127.7, 127.1, 125.6, 66.9, 48.6, 43.6, 21.8, 19.5; IR (KBr): *v*_max_ 3357, 3063, 3032, 2934, 1673, 1606, 1571, 1524, 1454, 1407, 1258, 1181, 1102, 1041, 810, 738 cm^−1^; HRMS (ESI+): *m*/*z* calcd for C_25_H_26_NO_3_ [M + H]^+^ 388.1907; found 388.1910.

Ethyl (1-(4-chlorophenyl)-3-oxo-3-(*p*-tolyl)propyl)carbamate (**18**): a white solid, mp: 109–110 °C. ^1^H NMR (400 MHz, CDCl_3_): *δ* 7.78 (d, J = 8.2 Hz, 2H), 7.31–7.26 (m, 4H), 7.23 (d, *J* = 8.2 Hz, 2H), 5.86 (br, 1H), 5.25 (m, 1H), 4.09 (q, *J* = 7.0 Hz, 2H), 3.72–3.53 (m, 1H), 3.39 (m, 1H), 2.39 (s, 3H), 1.21 (t, *J* = 7.0 Hz, 3H); ^13^C NMR (100 MHz, CDCl_3_): *δ* 197.5, 156.1, 144.6, 140.4, 134.3, 133.2, 129.6 (×2), 128.9 (×2), 128.4 (×2), 128.0 (×2), 61.2, 51.3, 43.8, 21.8, 14.7; IR (KBr): *v*_max_ 3345, 3033, 2980, 2928, 1688, 1607, 1531, 1492, 1444, 1410, 1367, 1335, 1257, 1182, 1091, 1047, 1014, 979, 813, 779, 759 cm^−1^; HRMS (ESI+): *m*/*z* calcd for C_19_H_21_ClNO_3_ [M + H]^+^ 346.1204; found 346.1206.

Ethyl (1-(2-chlorophenyl)-3-oxo-3-(*p*-tolyl)propyl)carbamate (**19**): a white solid, mp: 125–126 °C. ^1^H NMR (400 MHz, CDCl_3_): *δ* 7.79 (d, *J* = 8.1 Hz, 2H), 7.49 (d, *J* = 7.5 Hz, 1H), 7.32 (d, *J* = 7.5 Hz, 1H), 7.24–7.19 (m, 3H), 7.15 (dd, *J*_1_ = *J*_2_ = 7.5 Hz, 1H), 6.16 (br, 1H), 5.60 (m, 1H), 4.07 (q, *J* = 6.9 Hz, 2H), 3.68–3.54 (m, 1H), 3.47–3.37 (m, 1H), 2.38 (s, 3H), 1.20 (t, *J* = 6.9 Hz, 3H); ^13^C NMR (100 MHz, CDCl_3_): *δ* 198.0, 155.9, 144.5, 139.1, 134.3, 132.4, 129.9, 129.5 (×2), 128.7, 128.4 (×3), 127.2, 61.1, 49.8, 42.1, 21.7, 14.6; IR (KBr): *v*_max_ 3443, 2979, 1679, 1535, 1442, 1406, 1350, 1260, 1180, 1060, 805, 755 cm^−1^; HRMS (ESI+): *m*/*z* calcd for C_19_H_21_ClNO_3_ [M + H]^+^ 346.1204; found 346.1208.

Ethyl (1-(2-methoxyphenyl)-3-oxo-3-(*p*-tolyl)propyl)carbamate (**20**): a white solid, mp: 117–118 °C. ^1^H NMR (400 MHz, CDCl_3_): *δ* 7.81 (d, *J* = 8.1 Hz, 2H), 7.32 (d, *J* = 7.4 Hz, 1H), 7.25–7.17 (m, 3H), 6.90 (dd, *J*_1_ = *J*_2_ = 7.4 Hz, 1H), 6.85 (d, *J* = 7.4 Hz, 1H), 5.98 (br, 1H), 5.54–5.52 (m, 1H), 4.08 (q, *J* = 7.1 Hz, 2H), 3.88 (s, 3H), 3.54–3.40 (m, 2H), 2.38 (s, 3H), 1.21 (t, *J* = 7.1 Hz, 3H); ^13^C NMR (100 MHz, CDCl_3_): *δ* 198.0, 156.7, 156.0, 144.0, 134.6, 129.4 (×2), 129.3, 128.7 (×2), 128.5 (×2), 120.9, 110.9, 60.9, 55.5, 49.7, 43.6, 21.7, 14.7; IR (KBr): *v*_max_ 3442, 3063, 2934, 2837, 1719, 1681, 1606, 1493, 1463, 1439, 1408, 1368, 1289, 1245, 1181, 1107, 1048, 812, 754 cm^−1^; HRMS (ESI+): *m*/*z* calcd for C_20_H_24_NO_4_ [M + H]^+^ 342.1700; found 342.1701.

## 4. Conclusions

In summary, we developed a Hf(OTf)_4_-catalyzed one-pot method for the synthesis of β-carbamate-protected Mannich bases directly from the reaction of aldehydes, ketones, and carbamates. Compared to the previously reported catalytic systems, this new method only requires 2 mol% catalyst and affords a diversity of β-carbamate ketones under solvent-free conditions in excellent yields within only 2–4 h. Our experimental results based on sequential bimolecular reactions show that the Hf(OTf)_4_-catalyzed three-component reaction should not be categorized as a Mannich or Mannich-type reaction. The replacement of amine with much less reactive carbamate radically changes the reaction pathway to sequential aldol condensation and aza-Michael addition.

## Figures and Tables

**Table 1:** Catalytic effects of Group IVB transition metal Lewis acids on the three-component synthesis of **1** ^1^.

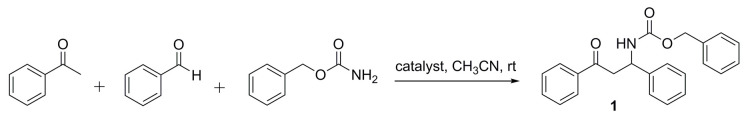			
Entry	Catalyst (10 mol%)	Reaction Time (h)	Isolated Yield (%)
1	Hf(OTf)_4_	18	71
2	HfCl_4_	24	55
3	Hf(acac)_4_	24	trace
4	HfCp_2_Cl_2_	24	trace
5	ZrCl_4_	24	trace
6	ZrCp_2_Cl_2_	24	trace
7	ZrOCl_2_	24	trace
8	Zr(NO_3_)_4_	24	n. r.
9	ZrO(NO_3_)_2_	24	n. r.
10	No catalyst	24	n. r.

^1^ Acetophenone/benzaldehyde/Cbz-NH_2_ 1:1:1.5 mmol ([benzaldehyde] = 0.5 M).

**Table 2:** Effects of solvent, temperature, and amount of catalyst loading on the three-component synthesis of **1** ^1^.

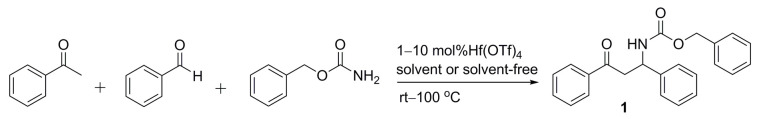					
Entry	Solvent	Catalyst (mol%)	Temperature (°C)	Reaction Time (h)	Isolated Yield (%)
1	CH_2_Cl_2_	10	20	48	52
2	Toluene	10	20	48	63
3	THF	10	20	48	67
4	DMF	10	20	24	n. r.
5	CH_3_CN	10	20	18	71
6	Solvent-free	10	20	8	82
7	Solvent-free	10	80	0.5	91
8	Solvent-free	10	100	0.33	83
9	Solvent-free	5	80	1	90
10	Solvent-free	2	80	2	90
11	Solvent-free	1	80	8	72
12	Solvent-free	2 (TfOH)	80	2	trace

^1^ Acetophenone/benzaldehyde/Cbz-NH_2_ 1:1:1.5 mmol ([benzaldehyde] = 0.5 M).

**Table 3:** Hf(OTf)_4_-catalyzed three-component synthesis of β-carbamate ketones **1**–**20**.

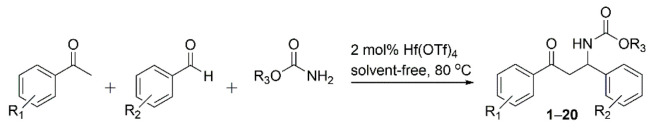
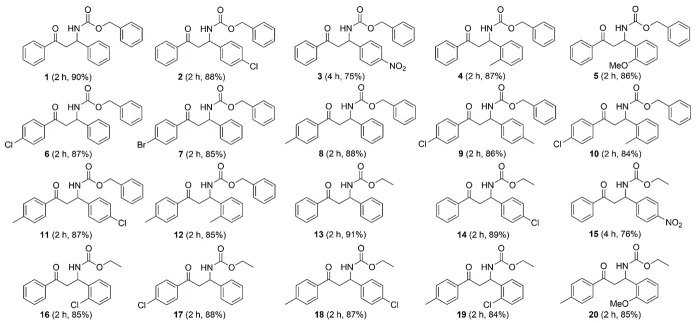

## Data Availability

The data presented in this study are available in Supplementary Materials.

## Supplementary Materials

The characterization data of known compounds and Figures S1–S20: The characterization spectra of new compounds [17,18,20,23,32,45].

## Sample Availability

Not available.
