# Supplementary material for: Hf(OTf)4-Catalyzed Three-Component Synthesis of N-Carbamate-Protected β-Amino Ketones
Source: Molecules. 2022 Feb 8;27(3):1122. doi: 10.3390/molecules27031122 (PMC8840004; doi:10.3390/molecules27031122)
Supplement: Supplementary file 1 [file molecules-27-01122-s001.zip › molecules-1572258-supplementary.pdf]

Supporting Information

Hf(OTf)$_4$-catalyzed three-component synthesis of *N*-carbamate-protected β-amino ketones

Zhen-Zhen Chen[1], Dong-Zhao Yang[1], Ying-Ying Dong[1], Mei Chi[1], Shou-Zhi Pu[1,2,*] and Qi Sun[1,*]

[1]*Jiangxi Key Laboratory of Organic Chemistry, Jiangxi Science and Technology Normal University, Nanchang, Jiangxi 330013, PR China*

[2]*Department of Ecology and Environment, Yuzhang Normal University, Nanchang, Jiangxi 330103, PR China*

Email: pushouzhi@tsinghua.org.cn (S.-Z.P.); sunqi@jxstnu.edu.cn (Q.S.)

Table of contents

1. Characterization data of known compounds **1**–**9**, **11**, and **13**–**17** Pages S2–S6

2. ^{1}H/^{13}C NMR, IR, and HRMS spectra of new compounds **10**, **12** and **18**–**20** (Figure S1–S20) Pages S7–S16

3. References Page S17

1. Characterization data of known compounds 1–9, 11, and 13–17

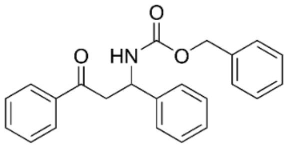

1

Benzyl (3-oxo-1,3-diphenylpropyl)carbamate (1): a white solid; mp 114–115°C; ^{1}H NMR (400 MHz, $CDCl_3$): *δ* 7.89 (d, *J* = 7.6 Hz, 2H), 7.56 (t, *J* = 7.4 Hz, 1H), 7.43 (t, *J* = 7.6 Hz, 2H), 7.36–7.22 (m, 10H), 5.91 (br, 1H), 5.43–5.29 (m 1H), 5.13–5.06 (m, 2H), 3.70–3.68 (m, 1H), 3.52–3.35 (m, 1H); ^{13}C NMR (100 MHz, $CDCl_3$): *δ* 198.0, 155.9, 141.5, 136.9, 136.6, 133.5, 128.8 (×4), 128.6 (×2), 128.3 (×3), 128.2 (×2), 127.7, 126.6 (×2), 67.0, 52.1, 44.2; IR (KBr): ν_{max} 3312, 3031, 2923, 1694, 1606, 1530, 1454, 1408, 1259, 1182, 1043, 812, 745 cm^{-1}; LRMS (ESI+): *m/z* calcd for $C_{23}H_{22}NO_3$ $[M+H]^+$ 360.2; found 360.2. [1,2]

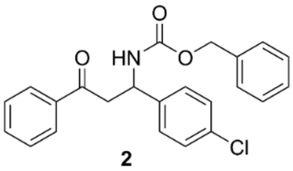

2

Benzyl (1-(4-chlorophenyl)-3-oxo-3-phenylpropyl)carbamate (2): a white solid; mp 120–121°C; ^{1}H NMR (400 MHz, $CDCl_3$): *δ* 7.86 (d, *J* = 7.6 Hz, 2H), 7.56 (t, *J* = 7.6 Hz, 1H), 7.41 (dd, $J_1 = J_2$ = 7.6 Hz, 2H), 7.36–7.24 (m, 9H), 6.01 (br, 1H), 5.29 (dt, $J_1 = J_2$ = 6.7 Hz, 1H), 5.13–5.04 (m, 2H), 3.68–3.60 (m, 1H), 3.46–3.38 (m, 1H); ^{13}C NMR (100 MHz, $CDCl_3$): *δ* 197.8, 155.7, 140.1, 136.6, 136.3, 133.6, 133.2, 128.8 (×4), 128.5 (×2), 128.1, (×4), 127.8 (×3), 66.9, 51.3, 43.8; IR (KBr): ν_{max} 3062, 3030, 2953, 1693, 1596, 1580, 1525, 1449, 1407, 1249, 1042, 1001, 748 cm^{-1}; LRMS (ESI+): *m/z* calcd for $C_{23}H_{21}ClNO_3$ $[M+H]^+$ 394.1; found 394.1.[1,2]

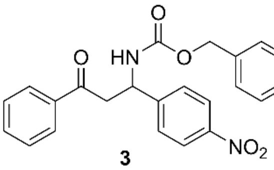

3

Benzyl (1-(4-nitrophenyl)-3-oxo-3-phenylpropyl)carbamate (3): a white solid; mp 143–144°C; ^{1}H NMR (400 MHz, $CDCl_3$): *δ* 8.17 (d, *J* = 8.4 Hz, 2H), 7.88 (d, *J* = 7.6 Hz, 2H), 7.59 (t, *J* = 7.6 Hz, 1H), 7.54 (d, *J* = 8.4 Hz, 2H), 7.45 (dd, $J_1 = J_2$ = 7.6 Hz, 2H), 7.40–7.29 (m, 5H), 6.15 (br, 1H), 5.45–5.33 (m, 1H), 5.13–5.09 (m, 2H), 3.83–3.66 (m, 1H), 3.59–3.41 (m, 1H); ^{13}C NMR (100 MHz, $CDCl_3$): *δ* 197.5, 155.9, 148.8, 147.0, 136.6, 136.3, 133.9, 128.8 (×4), 128.3 (×2), 128.2 (×4), 127.0, 123.8 (×2), 66.6, 52.1, 43.2; IR (KBr): ν_{max} 3030, 2949, 2843, 1704, 1637, 1530, 1500, 1472, 1315, 1270, 1208, 1181, 1048, 975, 884, 765 cm^{-1}; LRMS (ESI+): *m/z* calcd for $C_{23}H_{20}N_2O_5$ $[M+H]^+$ 405.1; found 405.1.[3]

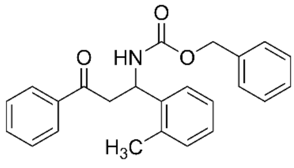

4

Benzyl (3-oxo-3-phenyl-1-(*o*-tolyl)propyl)carbamate (4): a white solid; mp 101–102 °C; ^{1}H NMR (400 MHz, $CDCl_3$): δ 7.89 (d, J = 7.7 Hz, 2H), 7.55 (t, J = 7.7 Hz, 1H), 7.43 (dd, J_1 = J_2 = 7.7 Hz, 2H), 7.36–7.33 (m, 6H), 7.15 (m, 3H), 5.56 (br, 2H), 5.11–5.03 (m, 2H), 3.64–3.60 (m, 1H), 3.48–3.45 (m, 1H), 2.45 (s, 3H); ^{13}C NMR (100 MHz, $CDCl_3$): δ 197.9, 155.7, 139.5, 136.9, 136.6, 135.8, 133.5 (×2), 131.0 (×2), 128.8 (×2), 128.7 (×2), 128.3 (×2), 127.7 (×2), 126.5, 125.6, 67.0, 48.6, 43.7, 19.5; IR (KBr): ν_{max} 3321, 3032, 2926, 1691, 1596, 1492, 1449, 1408, 1256, 1091, 1044, 830, 754 cm^{-1}; LRMS (ESI+): *m/z* calcd for $C_{24}H_{24}NO_3$ $[M+H]^+$ 374.2; found 374.2.[1]

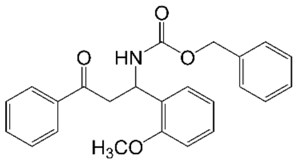

5

Benzyl (1-(2-methoxyphenyl)-3-oxo-3-phenylpropyl)carbamate (5): a white solid; mp 71–72 °C; ^{1}H NMR (400 MHz, $CDCl_3$): δ 7.91 (d, J = 7.6 Hz, 2H), 7.54 (t, J = 7.6 Hz, 1H), 7.42 (dd, J_1 = J_2 = 7.6 Hz, 2H), 7.38–7.35 (m, 6H), 7.23 (t, J = 7.9 Hz, 1H), 6.93 (t, J = 7.5 Hz, 1H), 6.87 (d, J = 8.2 Hz, 1H), 6.15 (br, 1H), 5.57–5.52 (m, 1H), 5.12–5.05 (m, 2H), 3.86 (s, 3H), 3.62–3.49 (m, 2H); ^{13}C NMR (100 MHz, $CDCl_3$): δ 198.3, 156.7, 155.7, 136.9, 136.7, 136.4, 133.2 (×2), 128.8 (×2), 128.7 (×2), 128.6 (×2), 128.3 (×2), 128.2 (×2), 120.9, 110.8, 66.8, 55.4, 49.8, 43.5; IR (KBr): ν_{max} 3431, 3063, 3033, 2955, 2838, 1687, 1599, 1493, 1448, 1401, 1340, 1245, 1048, 752 cm^{-1}; LRMS (ESI+): *m/z* calcd for $C_{24}H_{24}NO_4$ $[M+H]^+$ 390.2; found 390.2.[3]

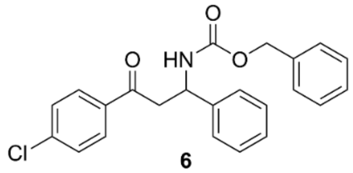

6

Benzyl (3-(4-chlorophenyl)-3-oxo-1-phenylpropyl)carbamate (6): a white solid; mp 98–99 °C; ^{1}H NMR (400 MHz, $CDCl_3$): δ 7.81 (d, J = 8.1 Hz, 2H), 7.39 (d, J = 8.4 Hz, 2H), 7.33–7.25 (m, 10H), 5.83 (br, 1H), 5.32 (dt, J_1 = J_2 = 6.1 Hz, 1H), 5.12–5.06 (m, 2H), 3.68 (br, 1H), 3.39 (dd, J_1 = 5.8 Hz, J_2 = 16.6 Hz, 1H); ^{13}C NMR (100 MHz, $CDCl_3$): δ 196.8, 155.8, 141.2, 140.1, 136.5, 135.1, 129.7 (×2), 129.1 (×2), 128.9, 128.7, 128.3 (×4), 127.8, 126.5 (×2), 67.0, 52.1, 44.2; IR (KBr): ν_{max} 3311, 3030, 2922, 1697, 1606, 1528, 1454, 1407, 1259, 1181, 1043, 812, 745 cm^{-1}; LRMS (ESI+): *m/z* calcd for $C_{23}H_{21}ClNO_3$ $[M+H]^+$ 394.1; found 394.1.[2]

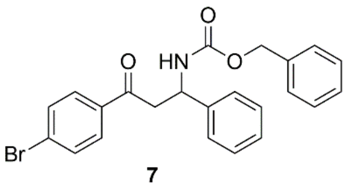

7

Benzyl (3-(4-bromophenyl)-3-oxo-1-phenylpropyl)carbamate (7): a white solid; mp 107–108 °C; ^{1}H NMR (400 MHz, $CDCl_3$): δ 7.71 (d, J = 8.0 Hz, 2H), 7.54 (d, J = 8.4 Hz, 2H), 7.26–7.09 (m, 10H), 5.86 (br, 1H), 5.38–5.24 (m, 1H), 5.09–5.00 (m, 2H), 3.70–3.58 (m, 1H), 3.31–3.17 (m, 1H); ^{13}C NMR (100 MHz, $CDCl_3$): δ 196.9, 155.8, 141.2, 136.5, 135.1, 132.1, 129.7 (×2), 128.9 (×2), 128.7, 128.6, 128.3 (×4), 127.7, 126.5 (×2), 67.0, 52.0, 44.2; IR (KBr): ν_{max} 3342, 3011, 2930, 1680, 1581, 1530, 1495, 1447, 1395, 1376, 1334, 1256, 1217, 1068, 1030, 829, 748 cm^{-1}; LRMS (ESI+): m/z calcd for $C_{23}H_{21}BrNO_3$ $[M+H]^+$ 437.1; found 437.1.[2]

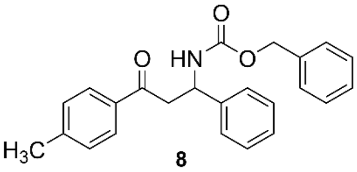

8

Benzyl (3-oxo-1-phenyl-3-(*p*-tolyl)propyl)carbamate (8): a white solid; mp 83–84 °C; ^{1}H NMR (400 MHz, $CDCl_3$): δ 7.76 (d, J = 8.0 Hz, 2H), 7.33–7.26 (m, 9H), 7.23–7.19 (t, J = 8.3 Hz, 3H), 5.95 (br, 1H), 5.31 (dd, $J_1 = J_2$ = 6.0 Hz, 1H), 5.09–5.03 (m, 2H), 3.68–3.59 (m, 1H), 3.46–3.29 (m, 1H), 2.36 (s, 3H); ^{13}C NMR (100 MHz, $CDCl_3$): δ 197.6, 155.8, 144.4, 141.6, 136.6, 134.3, 129.4 (×2), 128.7 (×2), 128.6 (×2), 128.3 (×2), 128.1 (×3), 127.5, 126.5 (×2), 66.9, 52.0, 43.9, 21.7; IR (KBr): ν_{max} 3319, 3031, 2922, 1691, 1606, 1528, 1454, 1407, 1259, 1181, 1043, 811, 745 cm^{-1}; LRMS (ESI+): m/z calcd for $C_{24}H_{24}NO_3$ $[M+H]^+$ 374.2; found 374.2.[2]

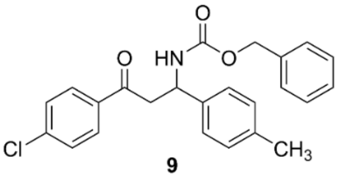

9

Benzyl (3-(4-chlorophenyl)-3-oxo-1-(*p*-tolyl)propyl)carbamate (9): a white solid; mp 135–136 °C; ^{1}H NMR (400 MHz, $CDCl_3$): δ 7.82 (d, J = 8.2 Hz, 2H), 7.40 (d, J = 8.2 Hz, 2H), 7.36–7.30 (m, 5H), 7.22 (d, J = 7.9 Hz, 2H), 7.12 (d, J = 7.9 Hz, 2H), 5.74 (br, 1H), 5.27 (m, 1H), 5.09 (m, 2H), 3.74–3.64 (m, 1H), 3.47–3.31 (m, 1H), 2.31 (s, 3H); ^{13}C NMR (100 MHz, $CDCl_3$): δ 196.9, 155.8, 140.0, 138.2, 137.5, 136.6, 135.2, 129.7 (×2), 129.6 (×2), 129.1 (×2), 128.8, 128.7, 128.3 (×2), 127.7, 126.5 (×2), 67.0, 51.9, 44.4, 21.2; IR (KBr): ν_{max} 3430, 1690, 1595, 1491, 1449, 1413, 1368, 1220, 1091, 1048, 1014, 830, 754 cm^{-1}; LRMS (ESI+): m/z calcd for $C_{24}H_{23}ClNO_3$ $[M+H]^+$ 408.1; found 408.1.[2]

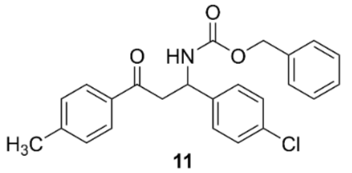

11

Benzyl (1-(4-chlorophenyl)-3-oxo-3-(*p*-tolyl)propyl)carbamate (11): a white solid; mp 125–126 °C; ^{1}H NMR (400 MHz, $CDCl_3$): δ 7.77 (d, J = 7.8 Hz, 2H), 7.37–7.22 (m, 11H), 6.01 (br, 1H), 5.29–5.27 (m, 1H), 5.10–5.09 (m, 2H), 3.68–3.62 (m, 1H), 3.42–3.37 (m, 1H), 2.39 (s, 3H); ^{13}C NMR (100 MHz, $CDCl_3$): δ 197.5, 155.9, 145.0, 144.7, 140.3, 136.5, 134.3, 133.3, 129.6 (×2), 128.9 (×2), 128.7 (×2), 128.4 (×4), 128.3, 128.0, 67.1, 51.6, 43.8, 21.8; IR (KBr): ν_{max} 3349, 1689, 1606, 1492, 1454, 1408, 1340, 1257, 1182, 1090, 1044, 1014, 812, 738 cm^{-1}; LRMS (ESI+): *m/z* calcd for $C_{24}H_{23}ClNO_3$ $[M+H]^+$ 408.1; found 408.1.[2]

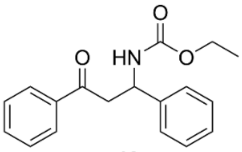

13

Ethyl (3-oxo-1,3-diphenylpropyl)carbamate (13): a white solid; mp 128–129 °C; ^{1}H NMR (400 MHz, $CDCl_3$): δ 7.90 (d, J = 7.6 Hz, 2H), 7.55 (t, J = 7.6 Hz, 1H), 7.43 (dd, $J_1 = J_2$ = 7.6 Hz, 2H), 7.36–7.21 (m, 5H), 5.83 (br, 1H), 5.34–5.29 (m, 1H), 4.09 (q, J = 7.0 Hz, 2H), 3.71–3.67 (m, 1H), 3.51–3.37 (m, 1H), 1.21 (m, 3H); ^{13}C NMR (100 MHz, $CDCl_3$): δ 198.0, 156.1, 141.6, 136.8, 133.5, 128.7 (×4), 128.4 (×2), 127.5, 126.5 (×2), 61.0, 51.8, 44.2, 14.6; IR (KBr): ν_{max} 3333, 3062, 3030, 2980, 1691, 1597, 1531, 1449, 1409, 1259, 1047, 1002, 871, 751 cm^{-1}; LRMS (ESI+): *m/z* calcd for $C_{18}H_{20}NO_3$ $[M+H]^+$ 298.1; found 298.1.[4,5]

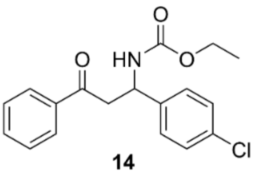

14

Ethyl (1-(4-chlorophenyl)-3-oxo-3-phenylpropyl)carbamate (14): a white solid; mp 112–113 °C; ^{1}H NMR (400 MHz, $CDCl_3$): δ 7.88 (d, J = 7.5 Hz, 2H), 7.55 (t, J = 7.5 Hz, 1H), 7.43 (dd, $J_1 = J_2$ = 7.5 Hz, 2H), 7.32–7.25 (m, 4H), 5.88 (br, 1H), 5.27 (m, 1H), 4.08 (q, J = 6.7 Hz, 2H), 3.68–3.64 (m, 1H), 3.49–3.36 (m, 1H), 1.20 (t, J = 6.7 Hz, 3H); ^{13}C NMR (100 MHz, $CDCl_3$): δ 197.8, 156.1, 140.3, 136.7, 133.7 (×2), 133.3, 128.8 (×3), 128.2 (×2), 128.0 (×2), 61.2, 51.3, 44.0, 14.7; IR (KBr): ν_{max} 3242, 2943, 1920, 1667, 1395, 1336, 993, 815, 796 cm^{-1}; LRMS (ESI+): *m/z* calcd for $C_{18}H_{19}ClNO_3$ $[M+H]^+$ 332.1; found 332.1.[6]

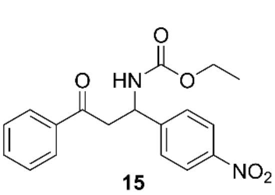

15

Ethyl (1-(4-nitrophenyl)-3-oxo-3-phenylpropyl)carbamate (15): a white solid; mp 133–134 °C; ^{1}H NMR (400 MHz, $CDCl_3$): δ 8.18 (d, J = 8.2 Hz, 2H), 7.88 (d, J = 7.2 Hz, 2H), 7.69–7.50 (m, 3H), 7.49–7.41 (m, 2H), 6.05 (br, 1H), 5.40–5.35 (m, 1H), 4.10 (q, J = 6.6 Hz, 2H), 3.75–3.64 (m, 1H), 3.50–3.41 (m, 1H), 1.21 (t, J = 6.6 Hz, 3H); ^{13}C NMR (100 MHz, $CDCl_3$): δ 197.7, 155.8, 148.8, 147.1, 136.3, 133.9, 128.7 (×4), 127.1 (×2), 123.9 (×2), 61.1, 51.8, 44.2, 14.6; IR (KBr): ν_{max}

3340, 2944, 1714, 1662, 1591, 1514, 1438, 1317, 1257, 1219, 1158, 1017, 983, 814, 751 cm^{-1}; LRMS (ESI+): *m/z* calcd for $C_{18}H_{18}N_2O_5$ $[M+H]^+$ 343.1; found 343.1.[4]

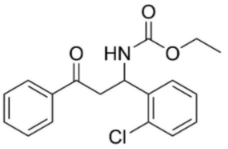

16

Ethyl (1-(2-chlorophenyl)-3-oxo-3-phenylpropyl)carbamate (16): a white solid; mp 128–129 °C; ^{1}H NMR (400 MHz, $CDCl_3$): δ 7.89 (d, J = 7.6 Hz, 2H), 7.54 (t, J = 7.6 Hz 1H), 7.50 (d, J = 7.6 Hz 1H), 7.41 (dd, J_1 = J_2 = 7.6 Hz, 2H), 7.33 (d, J = 7.6 Hz, 1H), 7.22 (dd, J_1 = J_2 = 7.6 Hz), 7.16 (dd, J_1 = J_2 = 7.6 Hz), 6.15 (br, 1H), 5.67–5.58 (m, 1H), 4.07 (q, J = 6.8 Hz, 2H), 3.76–3.57 (m, 1H), 3.54–3.41 (m, 1H), 1.32 (t, J = 6.8 Hz, 3H); ^{13}C NMR (100 MHz, $CDCl_3$): δ 198.4, 155.9, 139.0, 136.7, 133.6, 132.4, 130.0, 128.8 (×2), 128.3 (×4), 127.2, 61.2, 49.8, 42.2, 14.6; IR (KBr): ν_{max} 2923, 1683, 1597, 1580, 1495, 1449, 1406, 1326, 1210, 1158, 1092, 814, 750 cm^{-1}; LRMS (ESI+): *m/z* calcd for $C_{18}H_{19}ClNO_3$ $[M+H]^+$ 332.1; found 332.1.[4,5]

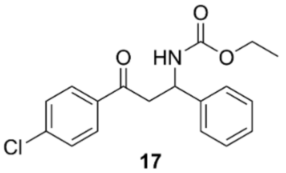

17

Ethyl (3-(4-chlorophenyl)-3-oxo-1-phenylpropyl)carbamate (17): a white solid; mp 114–115 °C; ^{1}H NMR (400 MHz, $CDCl_3$): δ 7.83 (d, J = 8.5 Hz, 2H), 7.40 (d, J = 8.5 Hz, 2H), 7.34–7.29 (m, 4H), 7.24 (m, 1H), 5.70 (br, 1H), 5.28 (m, 1H), 4.09 (q, J = 6.8 Hz, 2H), 3.69–3.65 (m, 1H), 3.47–3.34 (m, 1H), 1.21 (t, J = 6.8 Hz, 3H); ^{13}C NMR (100 MHz, $CDCl_3$): δ 196.8, 156.1, 141.4, 140.0, 135.2, 129.7 (×2), 129.1 (×2), 128.8 (×2), 127.7, 126.5 (×2), 61.2, 51.9, 44.3, 14.7; IR (KBr): ν_{max} 3337, 1690, 1589, 1570, 1530, 1401, 1369, 1260, 1174, 1092, 1048, 1012, 823, 761 cm^{-1}; LRMS (ESI+): *m/z* calcd for $C_{18}H_{19}ClNO_3$ $[M+H]^+$ 332.1; found 332.1.[3]

2. $^{1}H/^{13}C$ NMR, IR and HRMS spectra of new compounds 10, 12, and 18–20

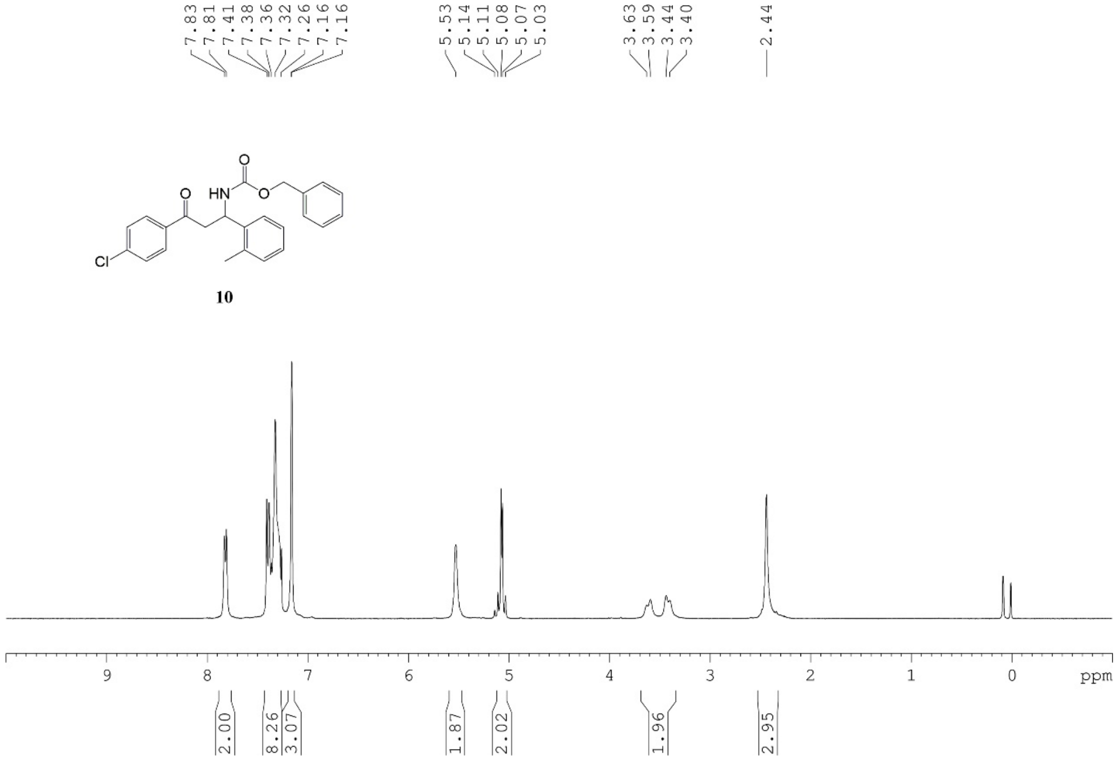

Figure S1. ^{1}H NMR spectrum of **10**

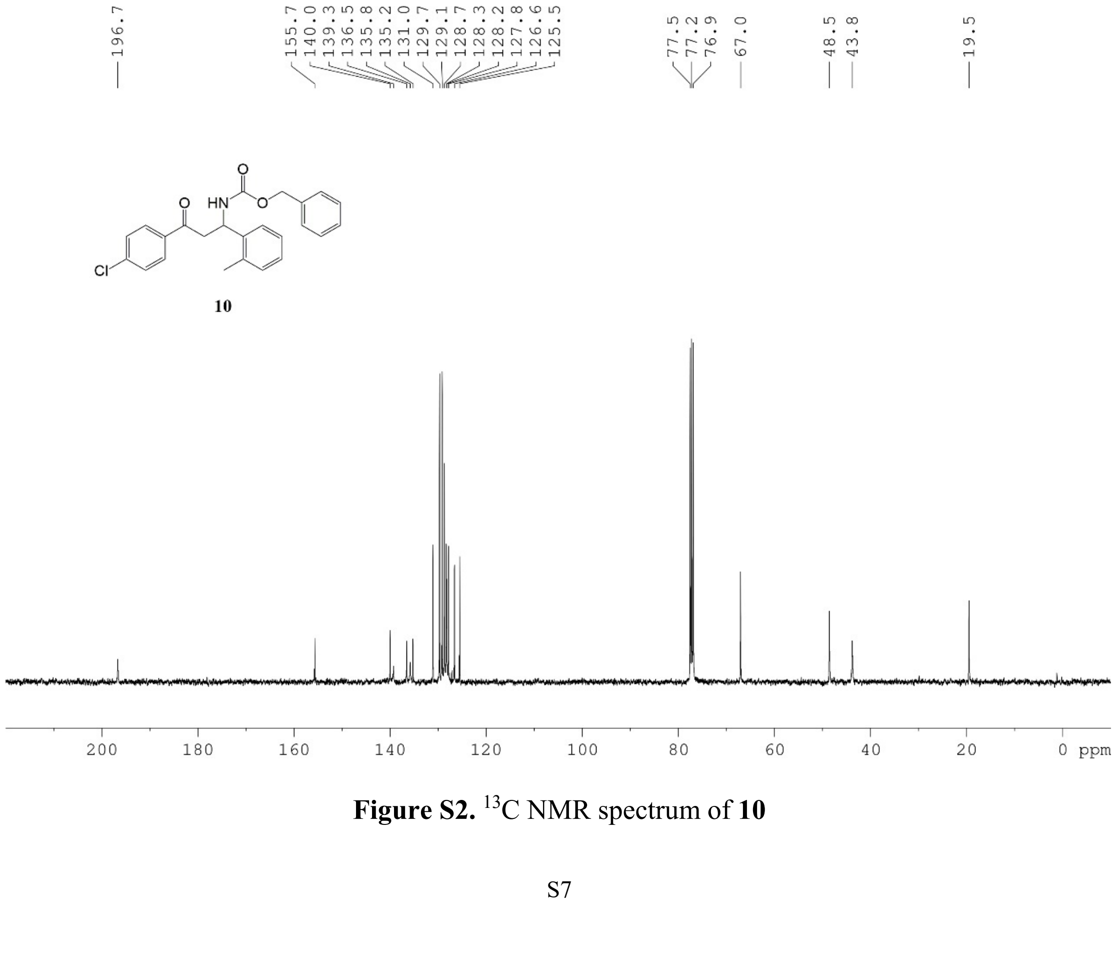

Figure S2. ^{13}C NMR spectrum of **10**

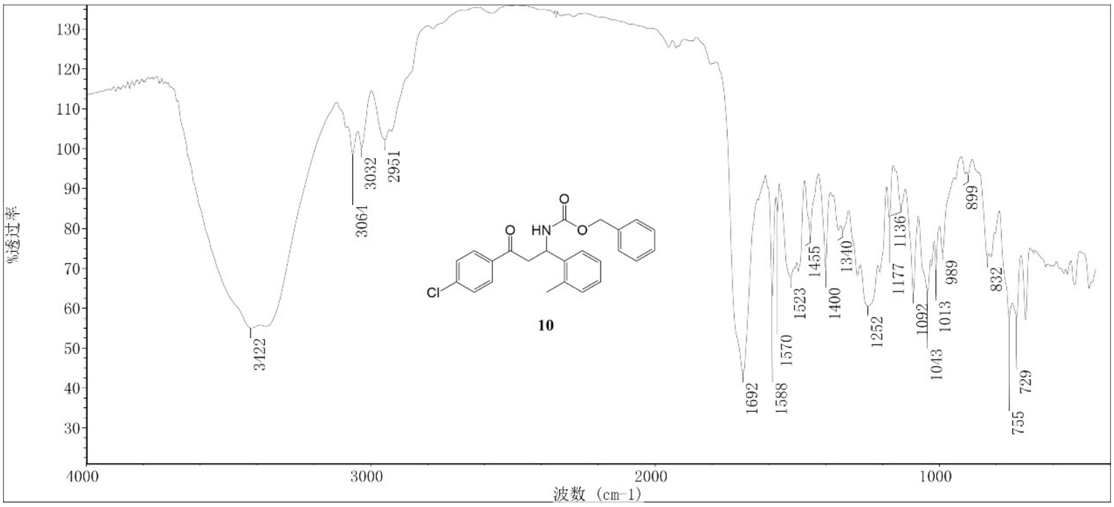

Figure S3. IR spectrum of **10**

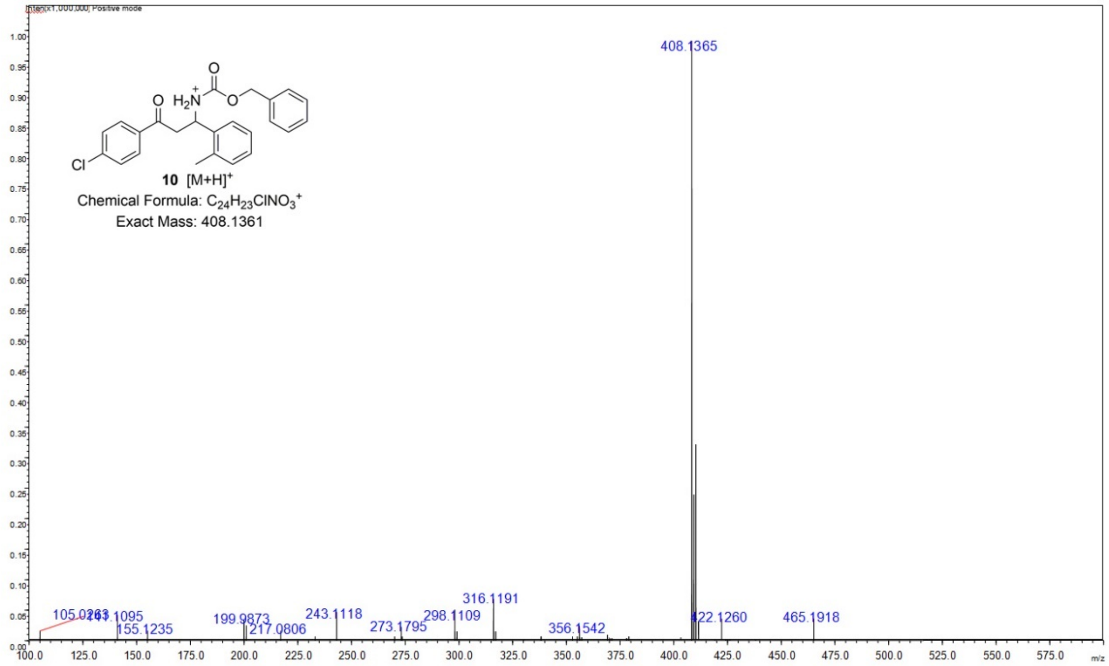

Figure S4. HRMS spectrum of **10**

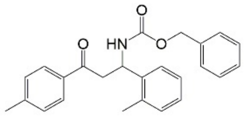

12

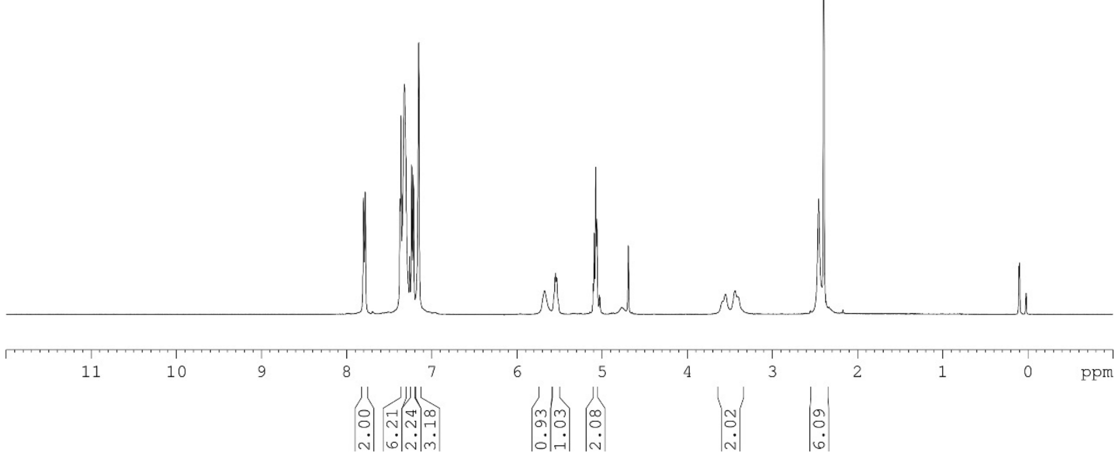

Figure S5. ^{1}H NMR spectrum of **12**

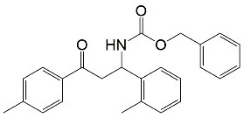

12

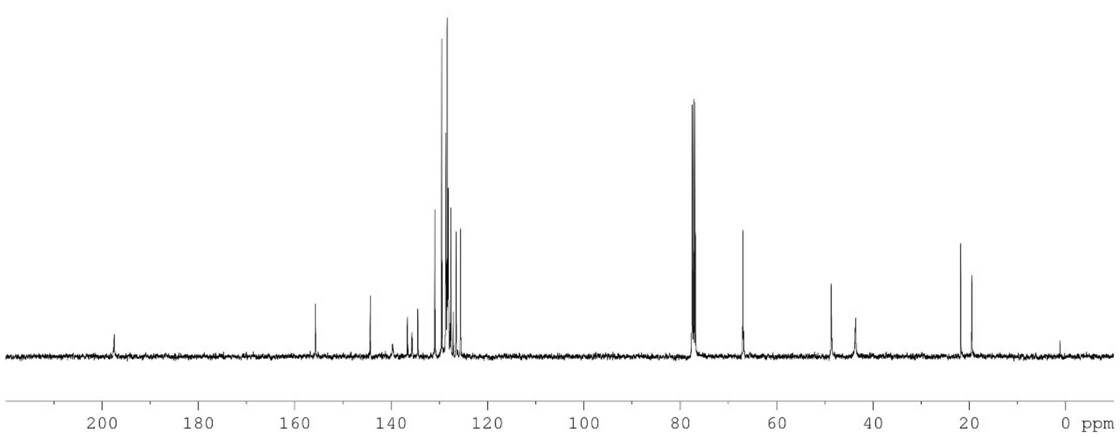

Figure S6. ^{13}C NMR spectrum of **12**

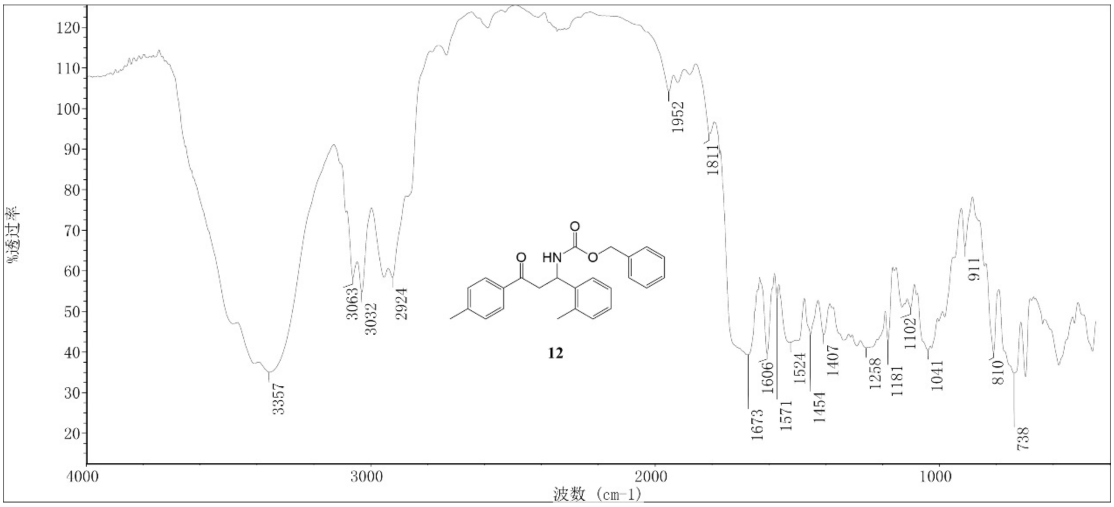

Figure S7. IR spectrum of **12**

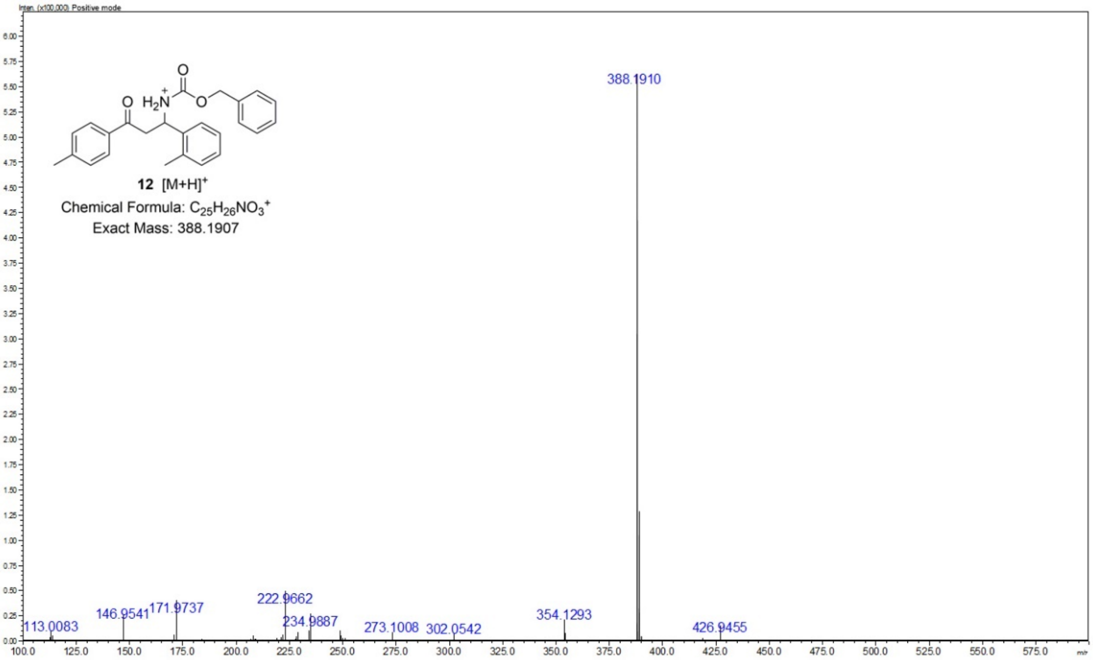

Figure S8. HRMS spectrum of **12**

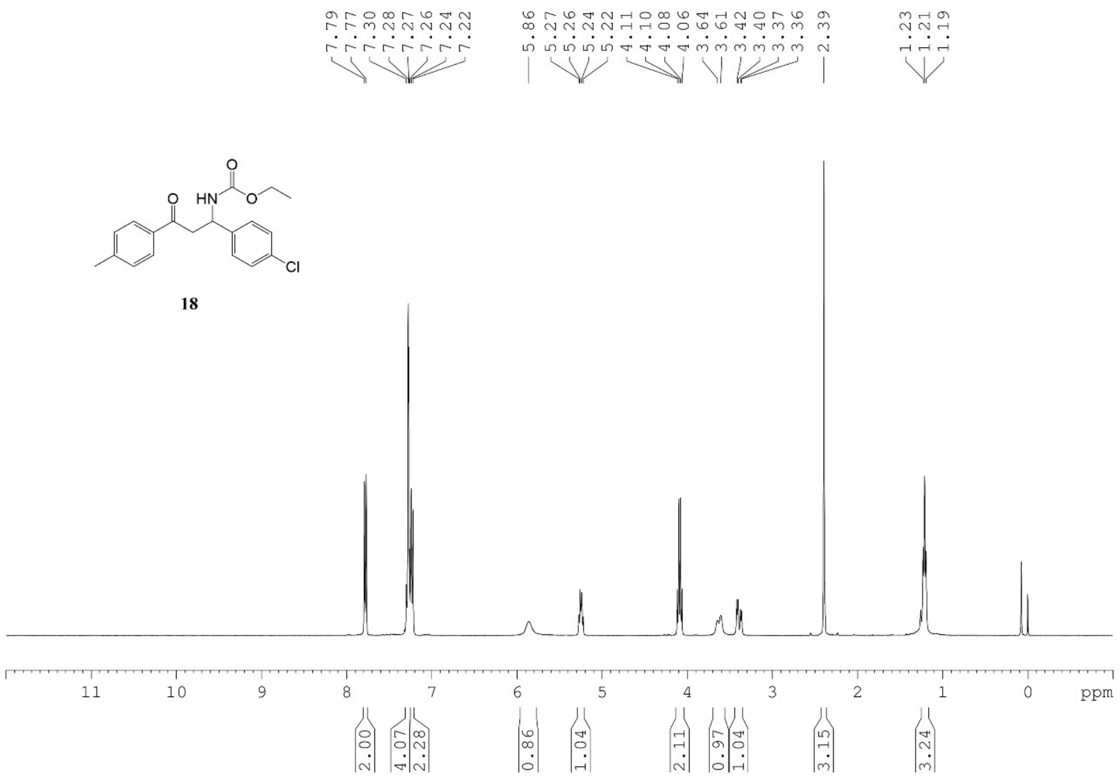

Figure S9. ^{1}H NMR spectrum of **18**

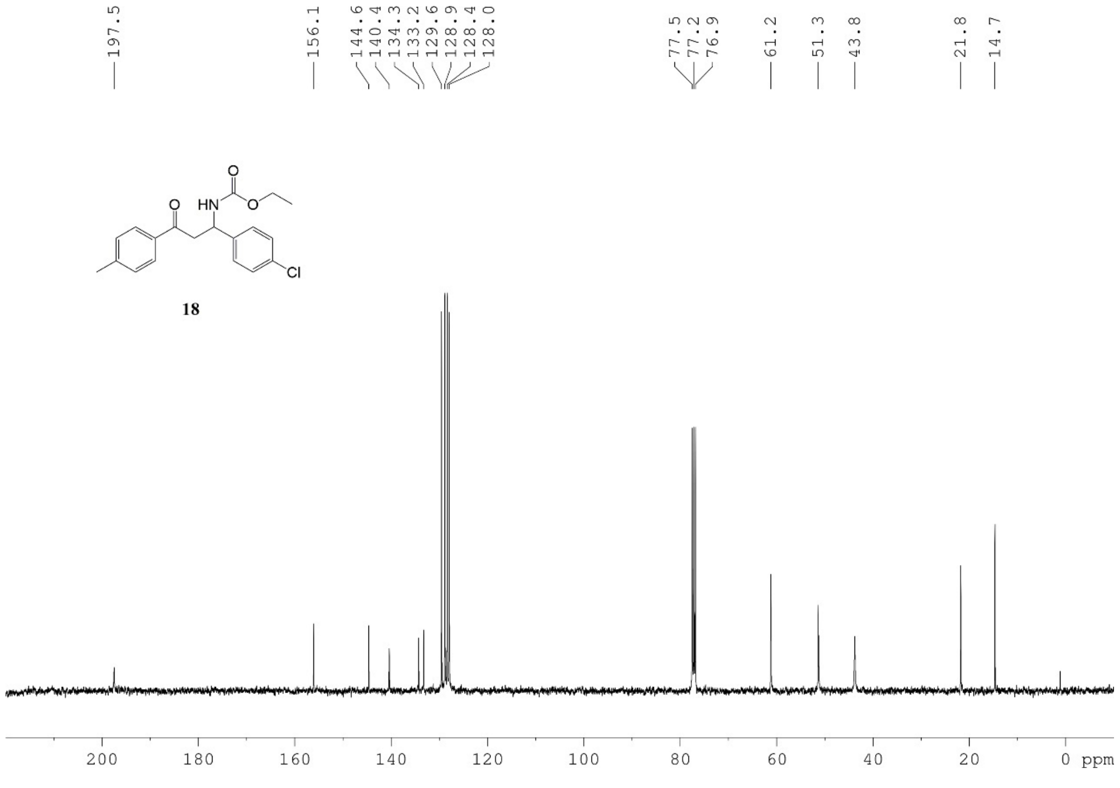

Figure S10. ^{13}C NMR spectrum of **18**

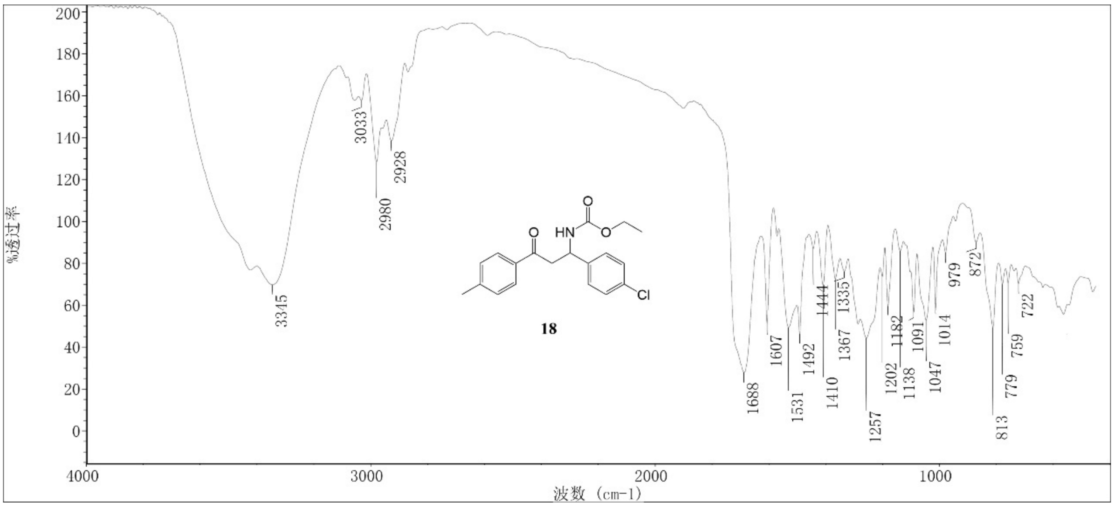

Figure S11. IR spectrum of **18**

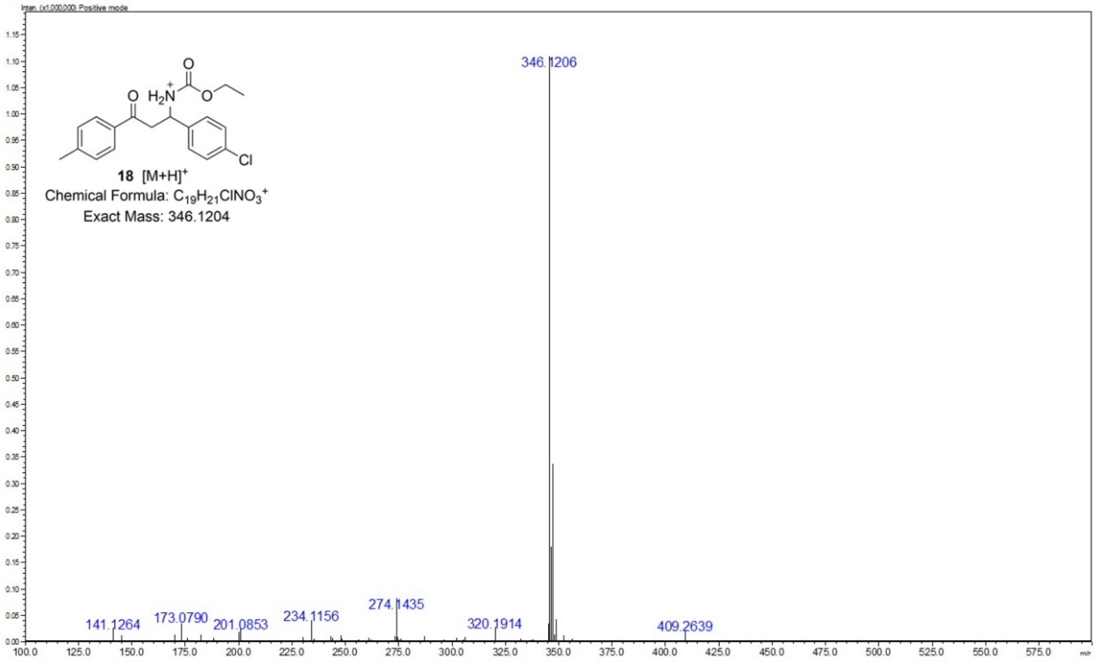

Figure S12. HRMS spectrum of **18**

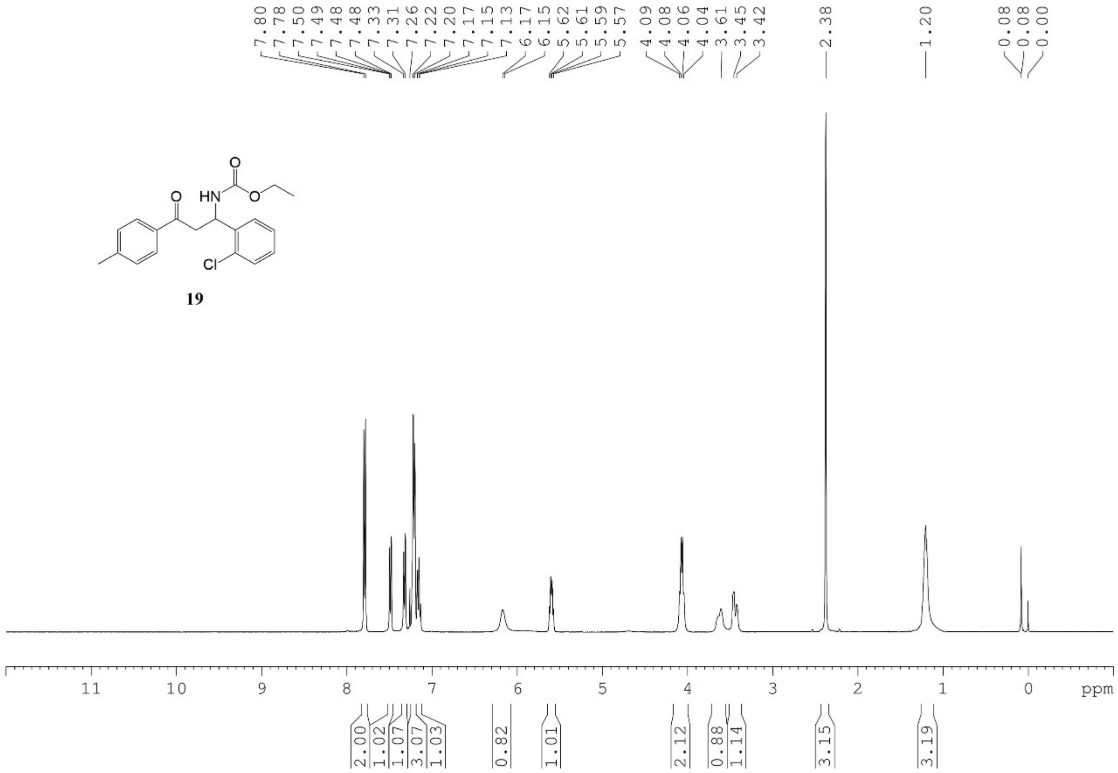

Figure S13. ^{1}H NMR spectrum of **19**

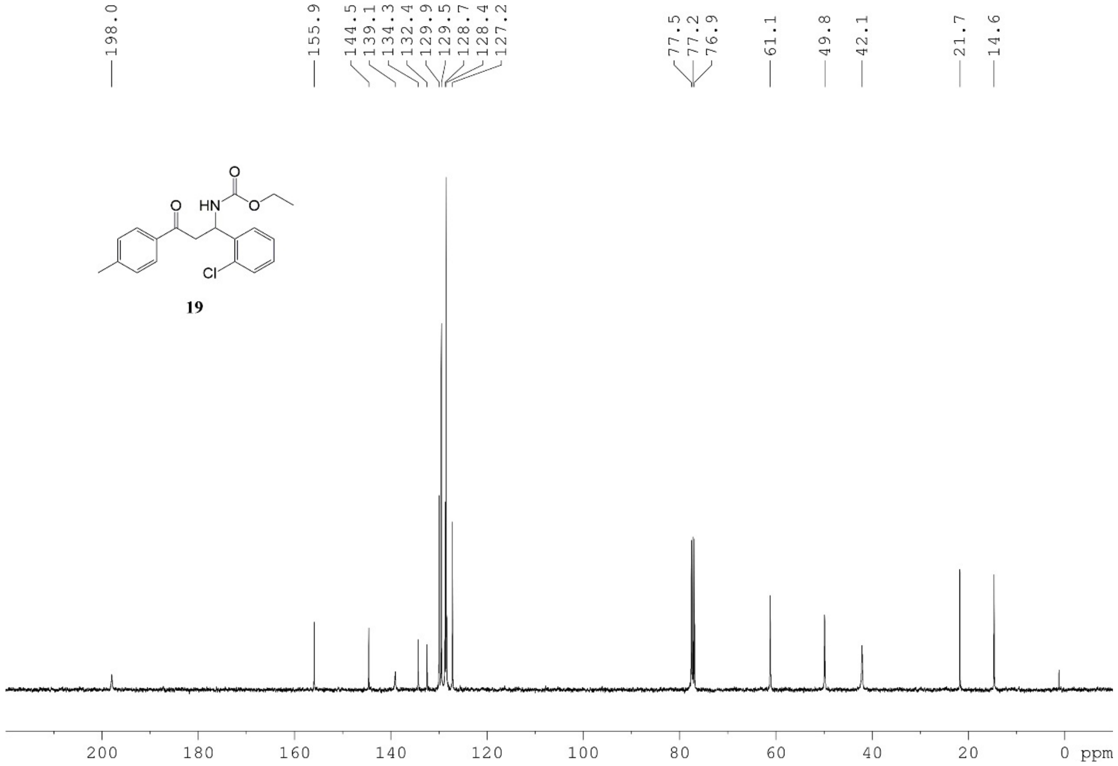

Figure S14. ^{13}C NMR spectrum of **19**

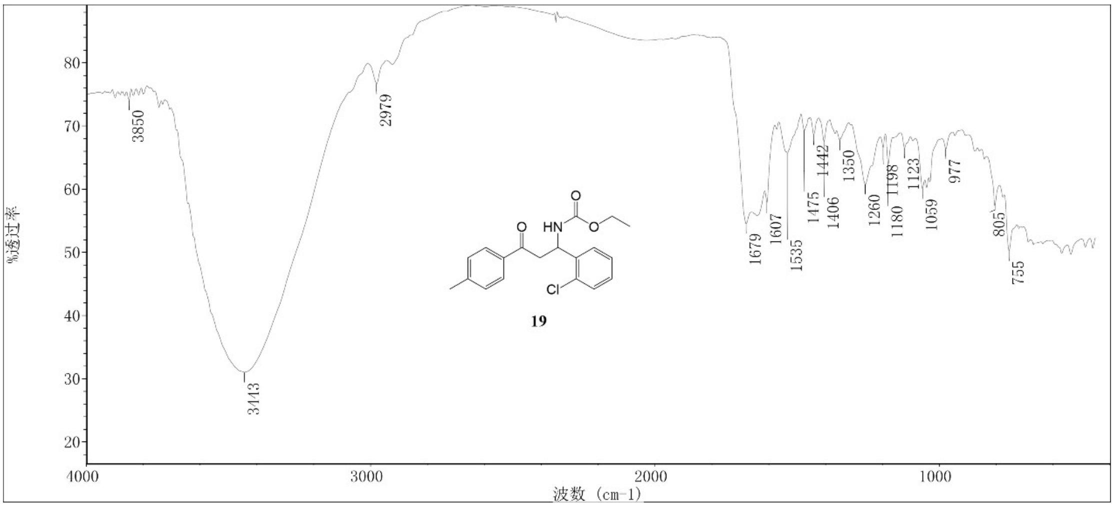

Figure S15. IR spectrum of **19**

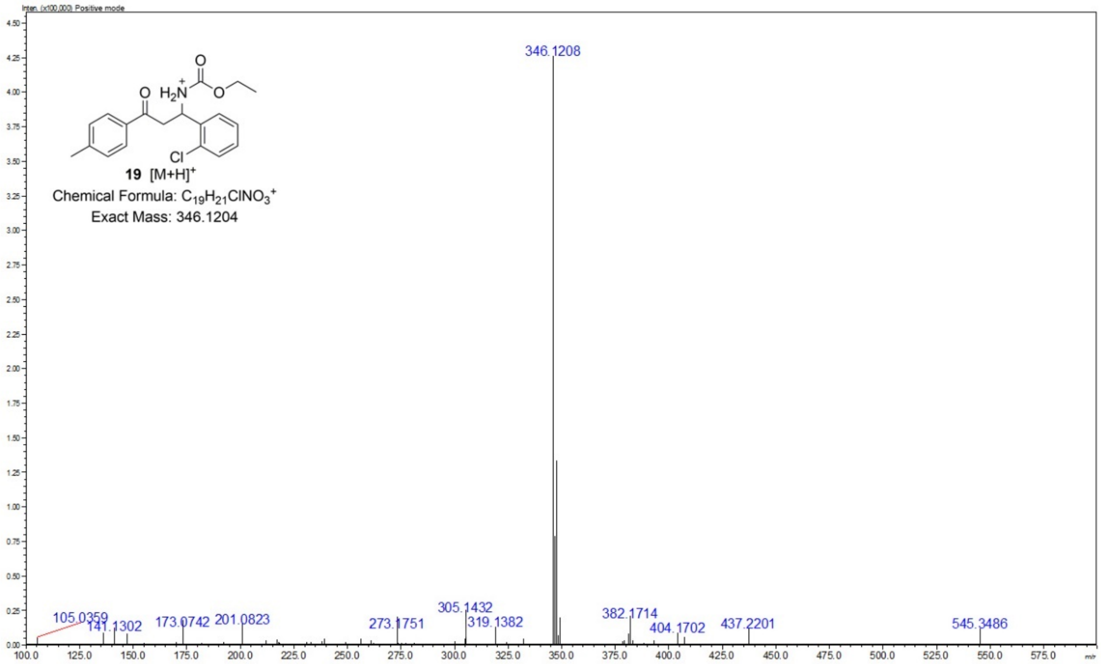

Figure S16. HRMS spectrum of **19**

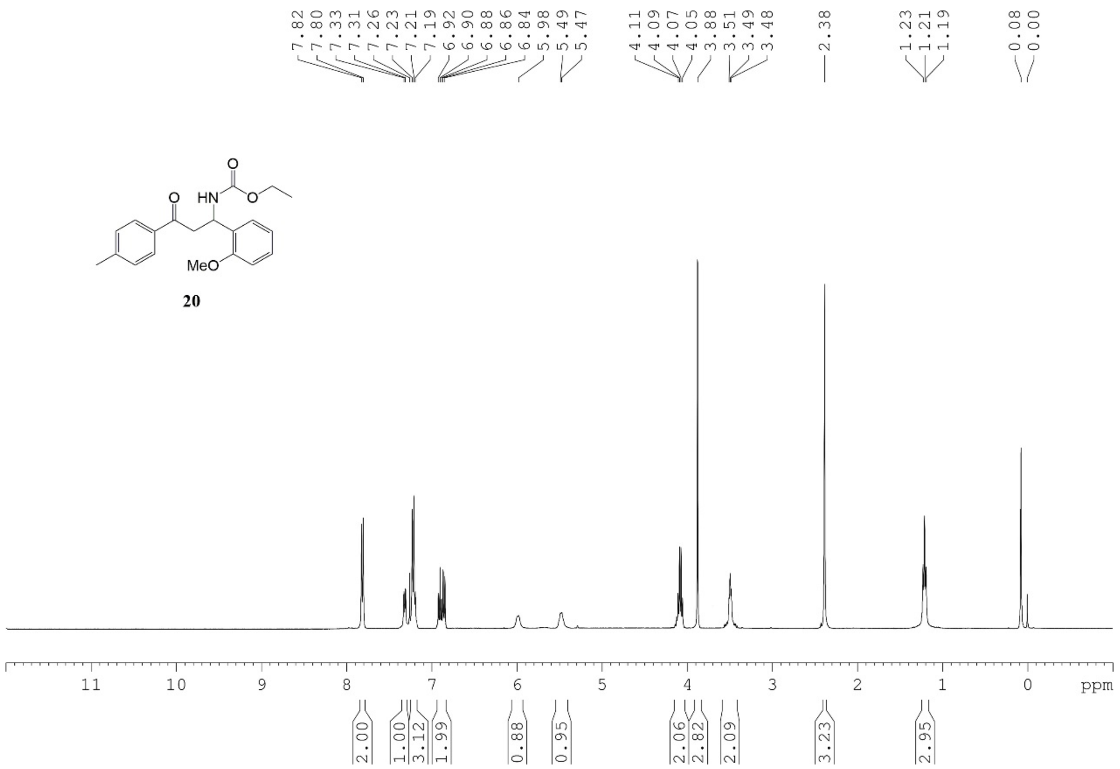

Figure S17. ^{1}H NMR spectrum of **20**

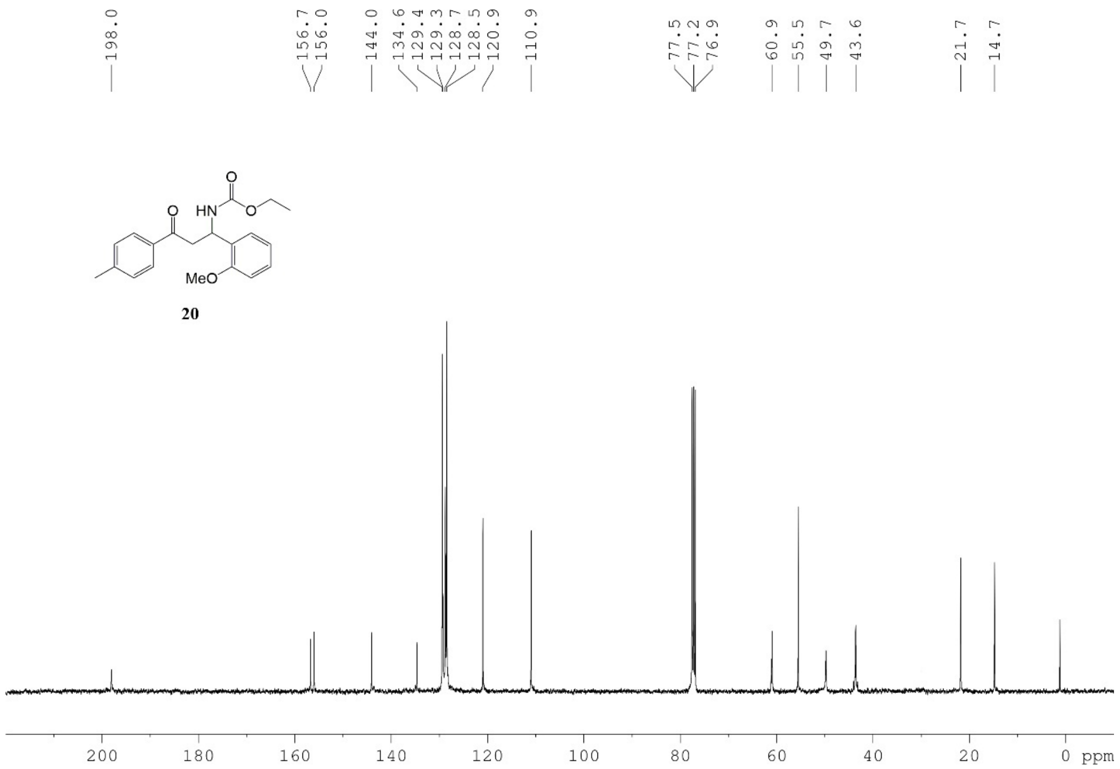

Figure S18. ^{13}C NMR spectrum of **20**

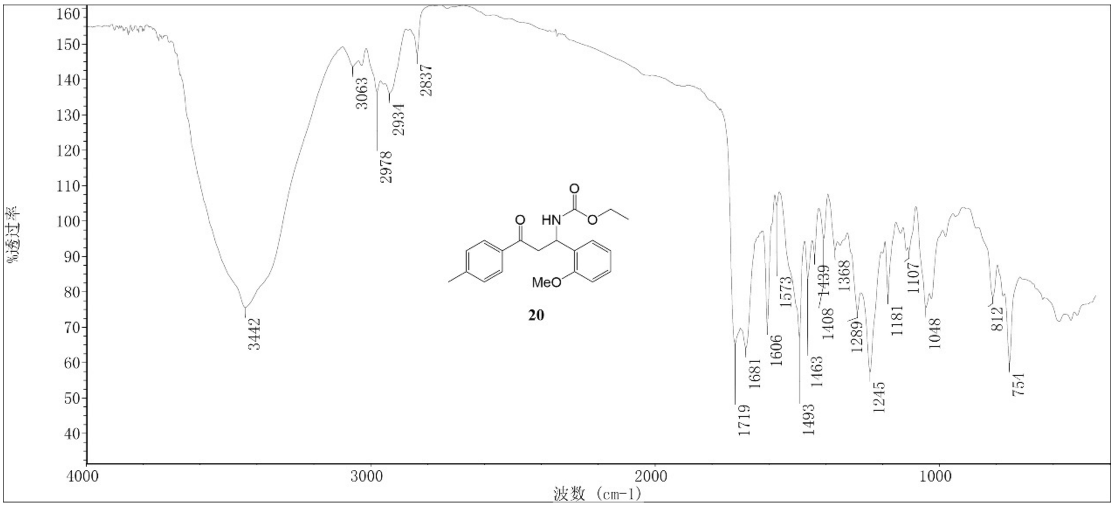

Figure S19. IR spectrum of **20**

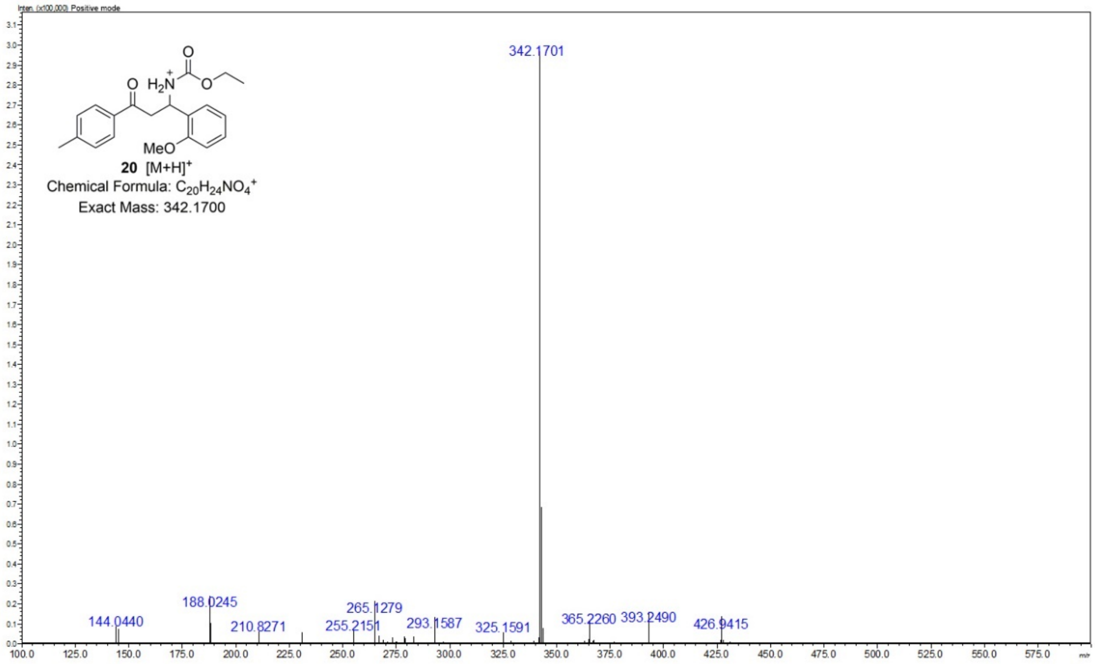

Figure S20. HRMS spectrum of **20**

3. References

1. Ollevier, T.; Nadeau, E.; Eguillona, J.C. The first catalytic Mannich-type reaction of *N*-alkoxycarbonylamino sulfones with silyl enolates. *Adv. Synth. Catal*. **2006**, *348*, 2080–2084.
2. Phukan, P.; Kataki, D.; Chakraborty, P. Direct synthesis of cbz-protected β-amino ketones by iodine-catalyzed three-component condensation of aldehydes, ketones and benzyl carbamate. *Tetrahedron Lett.* **2006**, *47*, 5523–5525.
3. Wang, R.; Huang, T.; Shi, L.; Li, B.-G.; Lu, X.-X. Heteropoly acids catalyzed direct Mannich reactions: Three-component synthesis of *N*-protected β-amino ketones. *Synlett* **2007**, *14*, 2197–2200.
4. Xu, L.-W.; L, L.; Xia, C.-G. TMSCl-Promoted transition metal-catalyzed aza-Michael reactions of chalcones with carbamates. *Synthesis* **2004**, *13*, 2191–2195.
5. Xu, L.-W.; Xia, C.-G.; Li, L. Transition metal salt-catalyzed direct three-component Mannich reactions of aldehydes, ketones, and carbamates: Efficient synthesis of *N*-protected β-aryl-β-amino ketone compounds. *J. Org. Chem.* **2004**, *69*, 8482–8484.
6. Fesenko, A.A.; Shutalev, A.D. Different modes of acid-catalyzed cyclization of 4-(γ-oxoalkyl) semicarbazide hydrazones: 7-membered versus 14-membered cyclic semicarbazones formation. *Tetrahedron* **2015**, *71*, 9528–9543.
